# Machine Learning in Relation to Emergency Medicine Clinical and Operational Scenarios: An Overview

**DOI:** 10.5811/westjem.2019.1.41244

**Published:** 2019-02-14

**Authors:** Sangil Lee, Nicholas M. Mohr, W. Nicholas Street, Prakash Nadkarni

**Affiliations:** * University of Iowa Carver College of Medicine, Department of Emergency Medicine, Iowa City, Iowa; † University of Iowa Carver College of Medicine, Department of Emergency Medicine, Anesthesia and Critical Care, Iowa City, Iowa; ‡ University of Iowa Tippie College of Business, Department of Management Sciences, Iowa City, Iowa; § University of Iowa Carver College of Medicine, Department of Internal Medicine, Iowa City, Iowa

## Abstract

Health informatics is a vital technology that holds great promise in the healthcare setting. We describe two prominent health informatics tools relevant to emergency care, as well as the historical background and the current state of informatics. We also identify recent research findings and practice changes. The recent advances in machine learning and natural language processing (NLP) are a prominent development in health informatics overall and relevant in emergency medicine (EM). A basic comprehension of machine-learning algorithms is the key to understand the recent usage of artificial intelligence in healthcare. We are using NLP more in clinical use for documentation. NLP has started to be used in research to identify clinically important diseases and conditions. Health informatics has the potential to benefit both healthcare providers and patients. We cover two powerful tools from health informatics for EM clinicians and researchers by describing the previous successes and challenges and conclude with their implications to emergency care.

## INTRODUCTION

Dr. Rob Procter, the editor of *Health Informatics Journal*, defined health informatics as “the interdisciplinary study of the design, development, adoption and application of information technology-based innovations in healthcare services delivery, management, and planning.^1^” The first digital computer was invented in the 1940s, and society was told that these new machines would soon be serving routinely as memory devices, assisting with calculations and information retrieval.^2^ Within the next decade, healthcare providers had started to benefit from the dramatic effects of this technology.^2^ Information technology has become so ingrained in modern medicine that contemporary practice depends on computational technology.

The recent advancement in health informatics has significant implications for biomedical research, as evidenced by searching PubMed for “Machine Learning” [Mesh] OR “Natural Language Processing” [Majr], which results in nearly 2400 publications related to machine learning and natural language processing (NLP) in 2017—up from 123 in 2010. This evolving technology also influences emergency care and research by aiding emergency medicine (EM) providers in several ways. First, it helps them to identify a high-risk condition by capturing data from available records or to prevent misdiagnoses by providing decision support.^3^ Second, it improves workflow efficiency by providing integrated decision aids within the electronic health record (EHR).^4^ Third, providers can maintain high-quality documentation in EHR in a high-tempo environment.^5^

We provide synopses of two clinically important health informatics applications: machine learning and NLP. These topics are diverse, and each of them is complex. Even in researching these areas, different researchers specialize in different sub-areas of machine learning or NLP. For example, speech-recognition and machine translation from one language to another are two distinct sub-fields with their own bodies of work. Machine learning and NLP are closely intertwined within the evolving healthcare system. Together, these health informatics applications offer many benefits to improve the practice of EM. The purpose of this review is for EM providers and researchers to understand two tools of health informatics, namely machine learning and NLP.

### Machine Learning

#### Definition

Machine learning is a computer science theory that often uses statistical techniques to give a computer, or artificial intelligence (AI), the ability to progressively improve performance on a given task based on the significant amount of data without any explicit program.^6^

#### Example

The use of machine learning has been integrated into our practice, for example, with automated white blood cell (WBC) differential count and computational electrocardiogram (ECG) analysis and interpretation.^7^–^9^ Recently, biomedical research findings using machine-learning algorithms were reported in mammograms for breast cancer screenings and retinal scans for diabetic retinopathy, wherein researchers used artificial neuron networks (ANN) and found higher sensitivity and specificity compared to an expert clinician panel.^10^,^11^ From EM literature, E-triage, a machine algorithm using random forest models, demonstrated superior predictability compared to the conventional Emergency Severity Index (ESI) triage.^12^

#### Basics of Machine Learning

Machine learning is designed to allow a program to infer patterns from three sets of data. First, the dataset used to adjust the weights on the learning algorithm (called the *classifier*) is the training set. The second data set is the *validation* data set. This dataset does not adjust the weights of the classifier but verifies that any increase in accuracy over the training dataset actually yields an increase in accuracy over a dataset that has not been shown to the classifier before, or at least the classifier hasn’t trained on it (i.e., validation). The third dataset is the *testing* set. This dataset is used only for testing the final solution in order to confirm the actual predictive power of the classifier.

### Glossary of Terms

Certain complex terminology associated with machine learning is described in this paragraph:

**Supervised learning**: These are input variables and output variables used to learn the mapping function from the input to output. In contrast, **unsupervised learning** does not provide any verification of output for the predictions. **Attribute** is a property or characteristic of an object that may vary, either from one object to another or from one time to another. Examples include the appearance of margins of a suspicious lesion in a chest radiograph and a suspected stroke in diffusion magnetic resonance imaging (MRI). **Class label** is a predefined category set as the goal to predict based on the attribute of computing the rules. Examples include abnormal ECGs, radiographs, and different types of WBCs in an automated differential count. **Classifier** is an algorithm that implements classifications, which involve the task of assigning objects to one of several predefined categories. Examples of classifiers include logistic regression, decision tree, and support vector machine (SVM). **Ensemble methods** constitute a combination of multiple, machine-learning algorithms to reduce the variance and bias and improve predictions.

#### Examples of Machine Learning

**Figure 1:**
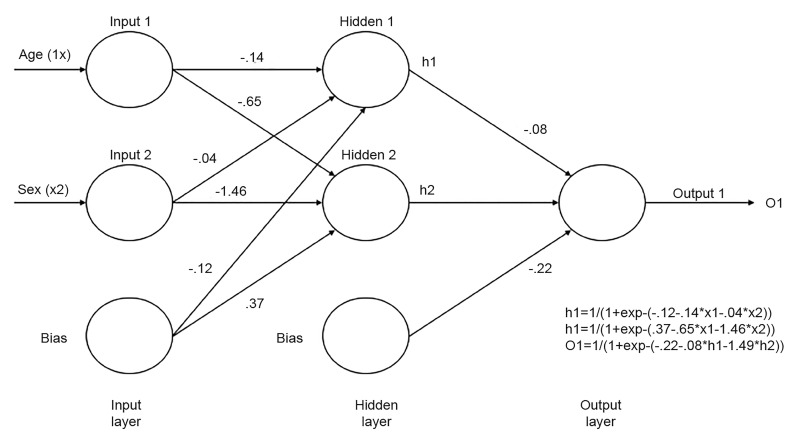
Diagram of artificial neuron networks.

The training process of the dataset is unique in each machine-learning algorithm (Table). One example of this is **artificial neuron networks** (ANN). In this case, each node functions as an artificial neuron and is connected to another node, and the connection has a weight to facilitate the learning process— similar to the human brain’s neuron network. The neurons are arranged in layers that function analogous to cells in the cerebral cortex and the retina. Figure 1 shows the diagram of an ANN that can predict the probability of a patient dying from a theoretical disease on the basis of the patient’s age (xl) and sex (x2). Each circle represents a node, while each line represents a connection weight. (Actual weight values are shown.) The nodes of the network are arranged in three layers (input, hidden, output). A logistic activation function is used in both the hidden (h1, h2) and output nodes (o1); (hl and h2 are the activations of hidden nodes 1 and 2; o1 is the predicted output of the network.) At each hidden and output node, a weighted linear combination of the inputs is summed and then a logistic transformation is applied.^13^

**Figure 2:**
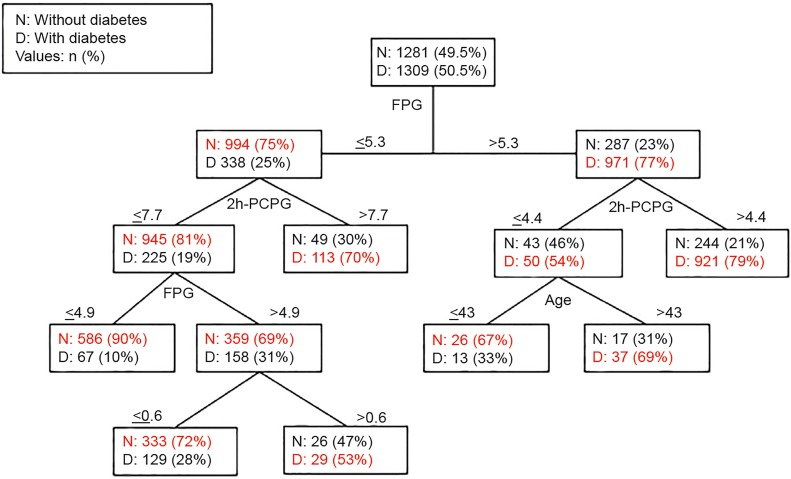
Diagram of decision tree. The original figure was created by Ramezankhani et al.^14^; the link is https://bmjopen.bmj.com/content/6/12/e013336.long. The shading and formatting of the lines between tree nodes and the text font are modified. *FPG*, Fasting Plasma Glucose; *PCPG*, Post Challenge Plasma Glucose.

A **decision tree** is an algorithm with a flowchart-like structure that recursively selects the best characteristics of an object to split the data (node) and expand the leaves. Figure 2 shows the example of decision trees for diabetes in men, including the predictor variables and the cut-off points for each predictor. It uses four variables (FPG = fasting plasma glucose, 2h-PCPG = 2-hour post-challenge plasma glucose, age and WHtR = waist-to-hip ratio) for classification and generated seven decision rules; each rule identifies a special subgroup with a certain probability of outcome (positive or negative) for each person in that subgroup. The FPG, located on the top of the tree, was the most important factor in the incidence of type 2 diabetes.^14^ A random forest model is a grouped or “ensemble” method designed to combine the predictions made by multiple decision trees (Table). Many of these models, especially the general linear model and regression and discriminant analysis, were invented long before the term “machine learning” was coined.^15^

#### Disadvantages of Machine Learning

**Figure 3:**
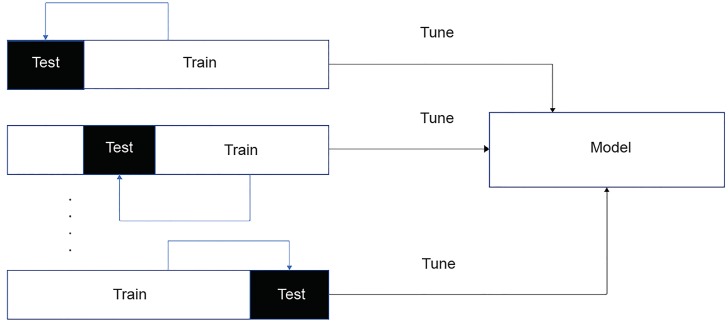
K-fold cross validation.* *The datasets are divided into several, equally sized subsets. The model is trained on subsets (training sets). After the training process, the model is tested on the remaining subsets (test sets). According to the number of subsets partitioned, user tests k-fold cross-validation. In *ten*-fold cross-validation, for example, one may use *10* results of *10*-fold cross-validation.

There are several issues with machine-learning algorithms to consider when we apply these clinically. One is its problem with **overfitting**, which is a potential problem with any prediction model. Overfitting occurs when the learning algorithm recognizes the false signal or noise in the dataset as the signal and applies the prediction to the test dataset, resulting in poor performance on new data or an inability to externally validate the model. There are several approaches to address this issue. One way to recognize overfitting is that the accuracy changes drastically, for example, the accuracy of 99% on the training set drops down to 50% when the algorithm is applied to the new dataset. If the accuracy over the training dataset increases, but the accuracy over the validation dataset stays the same or decreases, then we should stop training. A statistical method, the *goodness of fit* test, can measure how closely the model’s predicted values match the observed (true) values. Lastly, when we have several comparable algorithms, we can employ the simplest ones so that the added benefit of any complexity can be determined. This is the concept of **parsimony**, which favors a simpler model among others. The risk of overfitting can be minimized further by a sampling technique including cross-validation, which repeatedly partitions the example data randomly into training and validation sets to validate the model’s predictions internally. The process of data partitioning, training, and validation is repeated multiple times, and the validation results are averaged across the training cycles^24^ (Figure 3).

Another problem is that most clinicians and possibly some researchers are not made aware of what a machine-learning algorithm does to produce its output. A classic example is the study to explore the outcome of pneumonia-related hospitalization in the 1990s, in which asthma was reported as a protective factor against pneumonia in the study.^25^ Most clinicians would know from their experience that comorbid asthma is not a protective factor. In any learning algorithm (later defined as classifier), such as a simple/multiple or logistic regression, if an independent variable is strongly associated with the outcome/output variable, then there are four possible interpretations: 1) the predictor causes the outcome; 2) the outcome causes the predictor; 3) there is a common (unconsidered) variable that is associated with both the predictor and outcome variable; and 4) the association is coincidental. It is important to note that correlation does not imply causation. The study employed the neural network (Table), an algorithm that is known not to be intelligible or difficult to interpret by humans. With simultaneous analyses using the same dataset, the authors confirmed that those who had pneumonia and comorbid asthma were more likely to be admitted to the intensive care unit and to receive better care, which led to an improved pneumonia-related outcome. Although neural networks outperformed logistic regression (Table), a statistical model that is often believed to be the standard, the investigators concluded that the algorithm was too risky because the model was not intelligible.^26^,^27^

Miotto et al. published an article that showed the possibility of predicting future illnesses by applying a machine-learning algorithm to the EHR.^28^ The algorithm was unique as it did not require the verification of the prediction or use of unsupervised learning. Responding to this, Will Knight wrote in *The Dark Secret at the Heart of AI*, “no one really knows how the most advanced algorithms do what they do. That could be a problem.”^29^ The real problem is that the final state of the internals of a neural network (a set of connections between nodes and the weight and sign of each connection) is not very meaningful in terms of understandability to a human domain expert. By contrast, the output of regression techniques and most other techniques is human-interpretable in terms of identifying the variables of importance. The lesson is that the validity of the machine-learning algorithm and its findings requires a careful interpretation, with particular attention on overfitting and caution with these prediction models, particularly when there is no external validation for the method to test the portability of the developed learning algorithm to another set of data.

### Natural Language Processing (NLP)

#### Definition

NLP is an area of computer science and AI concerned with the interactions between computers and natural languages, particularly how to program computers to process and analyze large amounts of language data.^30^

#### Example

The advancement of NLP brought invaluable tools to clinical practice in day-to-day documentation, whether it is speech recognition software or macros and templates built into the EHR. Language modeling for automatic speech recognition uses a set of co-occurring words within a given window to find the most probable string of words out of candidate strings stored in the dataset.^31^ Word-error rate and accuracy rate are used to evaluate speech-recognition systems.^32^ Another example includes a statistical parser, which determines the most likely interpretation of a word or phrase in a sentence by using the conditional probability (a measure of the probability of an event given that another event has occurred).^33^ Recent studies have used NLP to identify diseases and conditions that are difficult to diagnose by clinical gestalt alone. For example, a study used NLP to detect Kawasaki disease based on the ED chart, implying its potential as decision support.^3^ Several other examples of using NLP for detection and prediction of disease and adverse events are reported in the literature.^34^–^38^

#### Development of NLP

NLP started in the 1950s as AI and linguistics crossed paths.^39^ Early-stage NLP, described as a word-for-word translation, was defeated by the problem of *homographs*, meaning identically spelled words with different meanings. For example, “minute” could mean time or small size depending on the context. The next tool, hand-written rules, faced NLP’s unrestricted volume and variations and encountered difficulty with extracting meaning from the text and poor handling of ungrammatical prose.^24^ Statistical NLP emerged as the result of reorientation as simple, robust approximations replaced deep analysis, evaluations became more rigorous, machine-learning methods using probabilities became prominent, and large, annotated bodies of text (corpora) were employed to train machine-learning algorithms— the annotation contained the correct answers and provided standards for evaluation.^40^ Machine learning is the core of NLP due to the unique features described above.^41^ Hand-written rules by humans have been largely replaced by machine learning for machine translation and speech recognition, and it is the driving force of contemporary NLP.^42^,^43^

#### Key Machine-learning Algorithms for NLP

Frequently used algorithms for natural NLP include the SVM and hidden Markov model (HMM). SVM classifies output such as words into categories that can be parts of speech from a variable or termed as a feature. The input is transformed to allow linear separation of the data points from different categories. Using the training data set, SVM identifies the hyperplane, a linearly separable boundary that divides the data into categories. HMM uses naïve Bayes, the algorithm based on the Bayes theorem (Table) as its core in that it applies conditional probability to sequential data (here, a sequence of words). HMM is the dominant algorithm used for speech recognition, which is used in radiology and pathology where semi-structured notes are used. The structure is imposed by templates, such as a chest radiograph report with normal findings in each system; and within each field, the contents are recorded as free text.

#### Real-world Application

Developing NLP algorithms to apply to real clinical problems is the next step in biomedical informatics. However, there are still crucial abstract data that cannot be acquired and stored in a way that allows for seamless decision-making practices. The text from a healthcare provider note is a great example of how data collection is influenced by provider-patient interactions. For example, a nurse may sense a patient’s anxiety and aim to achieve maximum cooperation while minimizing harm to the patient in real life, yet the data itself may not truly reflect the nuances of the interaction. Electronic documentation, particularly free text, may provide this information, but notes cannot be translated and incorporated into the data. Johnson et al. proposed switching to a “hybrid approach that combines semi-structured data entry and NLP within a standards-based and computer-processible document structure.”^44^ With the use of NLP, recent research focused on text-mining techniques (a type of data extraction method from sentences) and has been incorporated into machine learning to develop prediction models. The previous example regarding Kawasaki disease also demonstrates that NLP’s use of text from providers’ notes does not work well when notes are too vague.^3^

#### Future of Machine Learning in Emergency Medicine

The concept of AI is not new but has made drastic progress since machine-learning algorithms have been developed in recent years. Other than machine learning for prediction and NLP, the areas where the practice of EM can adopt these technologies include machine vision (such as automatic interpretation of imaging studies to screen and triage rapidly); the use of text-mining to facilitate public health surveillance through automated analyses of emergency department documents, and algorithm-based warning systems for cardiovascular or neurological decline. Intelligible machine learning holds a promise in improving the practice of EM.

#### Limitations of this Review

We used unstructured search methods for this narrative review of selected articles. Since these are based on the authors’ expertise, they may be biased.

## CONCLUSION

We described two important health informatics-related topics that are relevant to emergency care and research: machine learning and NLP. Traditionally, the machine-learning model in healthcare has suffered from low external validity or poor portability between sites, but this seems to be changing with active employment of creative solutions. NLP is highly problem-specific, and the tools available are intended for use by programmers rather than end-users, except for speech recognition and machine translation (the use of software to translate text or speech from one language to another). NLP is being used more for research purposes, but there is no general purpose information-extraction tool because what one chooses to extract depends on the problem one is trying to solve. Computational artifacts are complex and hinder our ability to predict the performance of these tools. It is important to carefully evaluate these tools using both subjective and objective approaches. It is prime time for clinicians and researchers in emergency medicine to take full advantage of health informatics to improve patient care.

## Figures and Tables

**Table:** Types and examples of machine-learning algorithms.

Study/year	Type of prediction model	Feature of model	Example
Nelder JA et al. 1972^15^	General linear model (GLM)	The technique used to obtain maximum likelihood estimates of the parameters with observations distributed according to some exponential family and systematic effects that can be made linear by a suitable transformation. A generalization of the analysis of variance is given for these models using log-likelihoods.^15^	The study aimed at forecasting daily emergency department (ED) visits using calendar variables and ambient temperature to compare the models in terms of forecasting accuracy.^16^
Lee E et al. 2012^17^	Discriminant analysis	A generalization of Fisher’s linear discriminant, a method used in statistics, pattern recognition, and machine learning, to find a linear combination of features that characterizes or separates two or more classes of objects or events.	This study was done to develop a clinical tool capable of identifying discriminatory characteristics that can predict patients who will return within 72 hours to the pediatric emergency department. The investigator used a classification model to predict return visits based on factors extracted from patient demographic information, chief complaint, diagnosis, treatment, and a hospital real-time ED statistics census.^17^
Lee S et al. 2017^18^	Logistic regression	A type of supervised learning that groups the variable to be predicted into classes (presence or absence of disease, for example) by estimating the probabilities with a logistic function. It is intelligible, meaning it is interpretable by humans.	To derive a prediction rule to stratify ED anaphylaxis patients at risk of a biphasic reaction, the authors conducted an observational study of a cohort of patients presenting to an academic ED with signs and symptoms of anaphylaxis. Logistic regression analyses were conducted to identify predictors of biphasic reactions, and odds ratios (ORs) are reported.^18^
Peck JS et al. 2012^19^	Naïve Bayes	A learning algorithm for binary (0 or 1) or categorical (1, 2, 3, 4, for example) problems. The calculations of the probabilities of each hypothesis are simplified to make their calculation tractable and choose the highest posterior probability (example: post-test probability) from the prior probabilities (example: pre-test probability). It is based on the strong assumption that the predictor variables do not interact and are conditionally independent of each other.	The objectives were to evaluate three models that use information gathered during triage to predict the number of ED patients that will subsequently be admitted to a hospital inpatient unit (IU) and to introduce a new methodology for implementing these predictions in the hospital setting. Three simple methods were compared with each other in order to predict hospital admissions at ED triage: expert opinion, naïve Bayes conditional probability, and a generalized linear regression model with a logit link function (logit-linear). Predictors considered included patient age, primary complaint, provider, designation (ED or fast track), arrival mode, and urgency level (emergency severity index assigned at triage).^19^
Hao S et al. 2014^20^	Decision tree	Decision tree is a flow chart–like structure in which each internal node denotes a test on an attribute, each branch represents the outcome of a test, and each leaf node holds a class label,^4^ which the model learns to predict. This algorithm works by recursively selecting the best attribute by which to split the node and expanding the leaf nodes of the tree until the stopping criterion is met.	A decision tree–based model with discriminant electronic medical record (EMR) features was developed and validated and estimated a patient ED 30-day revisit risk. A retrospective cohort was assembled with the associated patients’ demographic information and one-year clinical histories before the discharge date as the inputs.^20^
Levin S et al. 2017.12	Random forest	A type of ensemble method designed for decision tree classifiers, which combines the prediction made by multiple decision trees, where each tree is generated based on the values of an independent set of random vectors.^21^	E-triage used the random forest model applied to triage data that predicts the need for critical care, an emergency procedure, and inpatient hospitalization in parallel and translates risk to triage level designations.^12^
Son YJ et al. 2010^22^	Support vector machine (SVM)	A type of supervised learning models with associated learning algorithms that analyze data used for classifications and regression analysis. Given a set of training examples, each marked as belonging to one or the other of two categories, an SVM training algorithm builds a model that assigns new examples to one category or the other, making it a binary linear classifier. SVM is a representation of the examples as points in space, mapped so that the examples of the separate categories are divided by a clear gap that is as wide as possible. New examples are then mapped into that same space and predicted to belong to a category based on which side of the gap they fall.	A study aims to identify predictors of medication adherence in heart failure patients. The investigators applied SVM for data classification. For a given set of training data, each marked as belonging to one of two categories. An SVM training algorithm develops a model by finding a hyperplane, which classifies the given data as accurately as possible by maximizing the distance between two data clusters. Data about medication adherence were collected from patients at a university hospital through a self-reported questionnaire.^22^
Wu Y et al. 1993^23^	Neural network	Information processing that derives meaning from complicated or imprecise data. Each node, functioning as an artificial neuron, is connected to another node, and the connection has weight to facilitate the learning process based on input and output, similar to the brain’s neural network.	A study on developing a decision-making aid for radiologists in the analysis of mammographic data used an artificial neural network. The algorithm was trained based on the features extracted from experienced radiologists. The performance of the neural network was found to be higher than the average performance of the resident and staff physician alone, concluding that such networks may provide a potentially useful tool for distinguishing between benign and malignant lesions in mammograms.^23^
